# ETHNICITY-SPECIFIC EXTREME LONGEVITY VARIANTS IN A CONSORTIUM OF FOUR CENTENARIAN STUDIES

**DOI:** 10.1093/geroni/igac059.1741

**Published:** 2022-12-20

**Authors:** Harold Bae, Anastasia Gurinovich, Zeyuan Song, Cristina Giuliani, Marianne Nygaard, Nir Barzilai, Annibale Puca, Paola Sebastiani

**Affiliations:** Oregon State University, Corvallis, Oregon, United States; Tufts Medical Center, Boston, Massachusetts, United States; Boston University, Boston, Massachusetts, United States; University of Bologna, Bologna, Emilia-Romagna, Italy; University of Southern Denmark, Odense, Syddanmark, Denmark; Albert Einstein College of Medicine, Bronz, New York, United States; IRCCS Multimedica, Milan, Lombardia, Italy; Tufts Medical Center, Boston, Massachusetts, United States

## Abstract

It has been shown that some longevity variants including the APOE variants have ethnicity-specific effects on EL within European ethnicities. The goal of the present study is to identify genetic variants whose effects vary by ethnicity by conducting a genome-wide association study of extreme longevity (EL: defined as living past the age at which less than 1% individuals from the 1900 - 1920 birth year cohorts survived) that includes the SNP-by-ethnicity interaction terms using a consortium of four centenarian studies: the New England Centenarian Study, the Long Life Family Study, the Southern Italian Centenarian Study, and the Longevity Gene Project. We used the Uniform Manifold Approximation and Projection (UMAP), a non-linear dimension reduction technique, to identify distinct ethnic clusters. The UMAP analysis revealed four distinct ethnic groups (Danish, Italian, Ashkenazi Jewish, and middle European) in the aggregated data set with 2223 cases and 5673 controls. Using a mixed effects logistic model with SNP-by-ethnicity interaction terms, we found 29 loci, in which the test of any interaction effect produced p< 10-5. The results showed that some variants had ethnicity-specific effects on EL. We sought for replication in two independent studies of longevity: the Danish Longevity Study and the Italian Longevity Study. In the Italian Longevity Study, rs7907949 (PFKP), rs11667516 (intergenic: RAB11B;MARCHF2), rs13245505 (intergenic: ZC3HC1;KLHDC10) replicated with a nominal significance level of 0.05. In the Danish Longevity Study, rs79853795 (PLCB1) and rs4072601 (SLC39A11) replicated. Future drug development should account for ethnic-specific differences in the genetic effects for higher efficacy for more diverse populations.

